# Responses to crizotinib in a patient with c-ros oncogene 1, receptor tyrosine kinase-positive advanced lung adenocarcinoma: A case report

**DOI:** 10.3892/ol.2014.2571

**Published:** 2014-09-26

**Authors:** NA ZHANG, JIN-JI YANG, XU-CHAO ZHANG, ZHI XIE, BIN-CHAO WANG, HAI-YAN TU, BEN-YUAN JIANG, YI-LONG WU

**Affiliations:** 1 Guangdong Lung Cancer Institute, Guangdong General Hospital and Guangdong Academy of Medical Sciences, Guangzhou, Guangdong 510080, P.R. China; 2 Second Clinical Medial Committee, Southern Medical University, Guangzhou, Guangdong 510515, P.R. China

**Keywords:** non-small cell lung cancer, gene rearrangements, receptor tyrosine kinase 1, immunohistochemistry, crizotinib, partial responses

## Abstract

Rearrangements to the c-ros oncogene 1, receptor tyrosine kinase (*ROS1*) gene are reported in 1–2% of lung adenocarcinomas. These rearrangements are associated with a response to the small-molecule tyrosine kinase inhibitor crizotinib. *ROS1* rearrangements can be detected using fluorescence *in situ* hybridization (FISH), which is considered the gold standard technique in detecting *ROS1* rearrangements, and determining whether a patient would respond well to crizotinib treatment. However, FISH is an expensive and time-consuming assay, requiring specialized microscopy equipment and some level of technical expertise. The present report describes the case of a patient with advanced lung adenocarcinoma, who was identified to be negative for *ROS-1* rearrangements by FISH, but positive by immunohistochemistry (IHC). The health of the patient improved following treatment with crizotinib. These results indicate that IHC assay could be an alternative option for the detection of *ROS1* gene rearrangements.

## Introduction

C-ros oncogene 1, receptor tyrosine kinase (*ROS1*) gene rearrangements are reported in 1–2% of lung adenocarcinomas and are sensitive to the small-molecule tyrosine kinase inhibitor crizotinib (1,2). Fluorescent *in situ* hybridization (FISH) is the gold standard technique used for detecting ROS1-positive adenocarcinomas and determining whether the cancer would respond well to crizotinib treatment. However, FISH is an expensive and time-consuming assay, requiring specialized microscopy equipment and technical expertise (1). Currently there are no reports of the use of immunohistochemistry (IHC) in the detection of *ROS1* rearrangements in order to guide treatment of advanced non-small cell lung cancer (NSCLC). The present study reports the case of a patient with advanced lung adenocarcinoma who was ROS1-positive by IHC, but negative by FISH; the patient was determined to have had a partial response to crizotinib. Written informed consent was obtained from the patient’s family.

## Case report

A 36-year-old male presented to the Department of Oncology of Changzhou Tumor Hospital (Changzhou, China) with a persistent cough, which he had suffered with for two years, 1-day hemoptysis and shortness of breath. The patient was currently smoking 100 packets of cigarettes per year and had an otherwise unremarkable medical history.

A physical examination revealed decreased breath sounds in both lung fields, however no other signs were revealed. Positron emission tomography/computed tomography (CT) scans revealed a mass measuring 6.3×6.2 cm in the right middle lung lobe, and the maximum standardized uptake value (SUV_max_) was measured as 9.4, with atelectasis of the right lower lobe. There were multiple ground-glass opacities (GGOs) in both lungs, the largest of which was positioned in the right apicoposterior segmental lung lobe, which measured ~2.0×2.5 cm, and the SUV_max_ was 2.4. The supraclavicular lymph nodes were grossly enlarged on both sides. There was no metastasis observed in any other organ.

**Figure 1:**
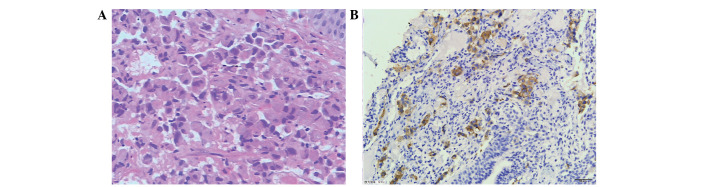
(A) Histological appearance of the bronchoscopic biopsy. Hematoxylin and eosin staining confirmed the diagnosis of adenocarcinoma. (B) Immunohistochemical analysis of c-ros oncogene tyrosine kinase receptor 1 protein expression. The staining intensity was measured as >70% 2+ or 3+ (magnification, ×20).

A fiber-optic bronchoscopy detected a stenotic lesion at the opening of the right middle lobe bronchus. A bronchoscopic biopsy confirmed the diagnosis of adenocarcinoma (Fig. 1A). Genotype analysis, using an amplification refractory mutations system, determined that the patient had expression of wild-type epidermal growth factor (*EGFR*), a commonly mutated oncogene. FISH and IHC showed that the patient was negative for the echinoderm microtubule-associated protein-like 4 (EML4)-anaplastic lymphoma kinase (ALK) fusion protein. The patient’s clinical stage was determined as T4(Ipsi_Nod_) N3M1a (contralateral lung) (stage IV).

**Figure 2:**
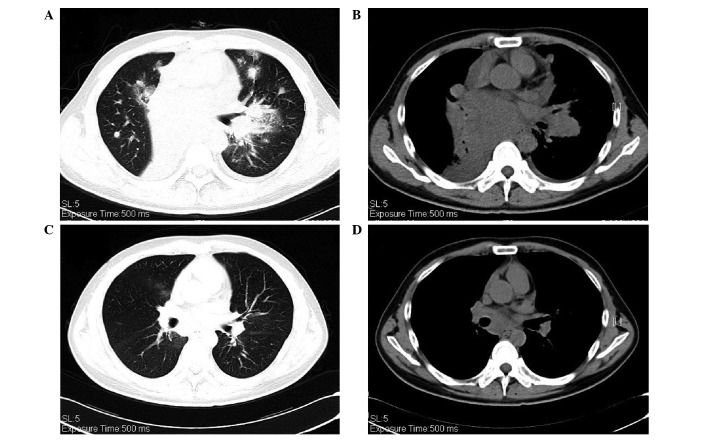
Computed tomography (CT) of the chest to observe the response to crizotinib treatment. CT scans of the chest were obtained (A and B) at baseline and (C and D) following 4 weeks of crizotinib treatment.

**Figure 3:**
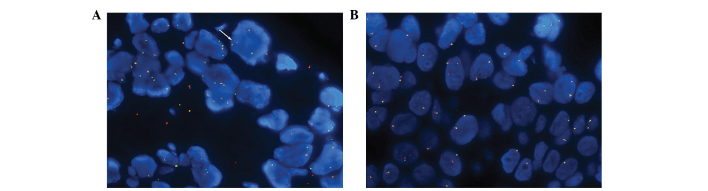
Fluorescence *in situ* hybridization analysis of c-ros oncogene tyrosine kinase receptor (*ROS1*) rearrangements. The adenocarcinoma was negative for *ROS1* rearrangements at a cutoff value of 15%. (A) Positive signal indicated (arrow; >15% of the cells demonstrate split or single 3′ signals). (B) Negative signal.

One cycle of chemotherapy was administered, containing pemetrexed (500 mg/m^2^, d1) and cisplatin (75 mg / m^2^, d1). One month later, the patient’s disease had progressed; the shortness of breath had worsened, and the pulmonary stenosis (PS) had deteriorated from 1 to 3. The CT scans showed that the targeted lesion in the right middle lobe had shrunk,; however, the nodules in the left lung had become enlarged, and there was a new lesion measuring ~1.0×1.3 cm at the hilus of the left lung. The presence of GGOs in both lungs had increased and enlarged (Fig. 2A and B). The ROS1 protein was detected using IHC, with a staining intensity of >70% 2+ or 3+ (Fig. 1B) (3). Furthermore, a break-apart FISH procedure was used to test for *ROS1* gene rearrangements; however, the result was negative when the cutoff value was set at 15% (Fig. 3A and B) (4).

The patient had severe PS and was being treated with no other targeted therapies, therefore crizotinib treatment was orally administered at the standard dose of 250 mg twice daily. One week later, the patient’s symptoms had improved and the PS was measured at 1, a decrease as compared with the previous measurement. Following four weeks of crizotinib treatment, the CT scans detected an improvement; the targeted lesions in the right lung and left lung hilus had shrunk and the GGOs had almost disappeared (Fig. 2C and D). According to the Response Evaluation Criteria in Solid Tumors criteria, version 1.1 (5), the patient’s cancer had elicited a partial response to the crizotinib treatment.

## Discussion

Alongside mutations in the *EGFR* gene and *EML4-ALK* rearrangements, *ROS1* rearrangements have been shown to define a new molecular subgroup in NSCLC (6). ROS1 shares a 49% amino acid sequence homology with EML4-ALK in the kinase domains, and several EML4-ALK inhibitors have demonstrated an inhibitory effect against ROS1 *in vitro* (7). Updated efficacy and safety data have been presented for the use of crizotinib in patients with advanced *ROS1* rearrangements in NSCLC (8). An ongoing phase I clinical trial, investigating the effects of crizotinib (NCT00585195), enrolled patients with advanced *ROS1*-rearranged NSCLC, as determined by FISH. The patients receive a standard oral dose of crizotinib: 250 mg twice daily. As observed in *EML4-ALK*-positive NSCLC, treatment with crizotinib has so far shown promising antitumor activity, with an overall response rate of 56% (2).

FISH is an expensive and difficult assay to support, requiring specialized microscopy equipment and technical expertise. By contrast, IHC is relatively cheap in comparison and can be performed in numerous pathology laboratories (1,9). A previous study showed that if IHC thresholds of 2+ and 3+ are considered to indicate positive expression, ROS1 IHC is 100% sensitive and 92% specific for *ROS1* rearrangements with FISH (1).

To the best of our knowledge, the present case report is the first to show positive ROS1-testing by IHC, but negative by FISH where the patient responded well to crizotinib treatment. These results provide evidence that IHC may be a useful screening method for the detection of *ROS1* rearrangements, thus allowing for targeted precision therapy.
